# Predicting Seizure Risk from Routine Electroencephalographs in Medical Intensive Care Units Using the 2HELPS2B Score

**DOI:** 10.3390/life15091455

**Published:** 2025-09-17

**Authors:** Cheng-Lun Hsiao, Wan-Ling Chang, Pei-Ya Chen, I-An Chen, Shinn-Kuang Lin

**Affiliations:** 1 Stroke Center and Department of Neurology, Taipei Tzu Chi Hospital, Buddhist Tzu Chi Medical Foundation, New Taipei City 23142,Taiwan; shb@ms19.hinet.net (C.-L.H.); laetitia0717@hotmail.com (W.-L.C.); ruentw@gmail.com (P.-Y.C.); 2 School of Medicine, Tzu Chi University, Hualien 97004, Taiwan; 3 Taiwan Center for Drug Evaluation, Regulatory Science Center of Consultation, Taipei 11557, Taiwan; iachen272@cde.org.tw

**Keywords:** 2HELPS2B score, antiseizure medications, critically illness, intensive care unit, routine electroencephalography, seizure

## Abstract

This study evaluated the utility of the 2HELPS2B score in predicting seizures from routine electroencephalographs (rEEGs). In total, 670 rEEGs obtained in a medical intensive care unit (MICU) between October 2018 and March 2023 were analyzed. More than 75% of these rEEGs were requested due to seizures and unexplained altered consciousness. Seizures occurred most frequently in patients with rEEGs characterized by brief, potentially ictal rhythmic discharges and electrographic seizures. A history of seizures was the most prevalent risk factor identified by the 2HELPS2B score. Seizures occurred in 28% of the cohort who experienced a seizure within 24 h of the rEEG and in 38% of the cohort who experienced a seizure before MICU discharge. Among the patients with suspected altered consciousness, the seizure incidence before MICU discharge (9.2%) was twice that within 24 h of the initial rEEG (4.7%). The seizure rate also increased from 12% for a 2HELPS2B score of 1 to 100% for scores ≥ 4. A score ≥ 2 was the optimal cutoff for predicting post-rEEG seizures and guiding antiseizure medication (ASM) treatment. Seizures occurred most frequently in patients whose ASMs were supplemented with new medications, and most new prescriptions for antiseizure medication were issued to patients with altered consciousness. These results demonstrate that the 2HELPS2B score can effectively predict seizures on the basis of rEEG results.

## 1. Introduction

Electroencephalographs (EEGs) are critical in evaluating the causes of altered mental states and abnormal behavior in intensive care settings [1]. Research recommends the use of continuous electroencephalographs (cEEGs) in intensive care units (ICUs), particularly for patients with altered consciousness [2]. Clinical applications of cEEGs include monitoring status epilepticus, evaluating the effects of seizure treatment, and detecting brain ischemia [3]. cEEGs can be used to identify nonconvulsive seizures (NCSs) and enhance outcomes in critically ill patients [4,5]. The American Clinical Neurophysiology Society (ACNS) has established standardized terminology for critical care EEGs [6]. The 2HELPS2B score was developed on the basis of ACNS standards and assesses seizure risk in patients in ICUs undergoing cEEGs [7]. This score evaluates six items used to assess patients’ seizure history and five distinct EEG patterns. The 2HELPS2B score has been validated for its ability to predict seizures such as NCSs and nonconvulsive status epilepticus (NCSE) in patients with critical illness or those suffering from acute cerebral injury in the emergency department or ICU [8,9,10,11]. Higher scores correspond to increased brain instability and a greater probability of imminent seizures. Although the 2HELPS2B score is highly clinically valuable, cEEGs remain underutilized in developed countries due to limitations in personnel and financing [12]. In clinical practice, however, routine EEGs (rEEGs) are more widely available than cEEGs, particularly in institutions where technical and financial resources for long-term monitoring are limited. rEEGs typically last 20–30 min and are commonly performed in the ICU to evaluate unexplained altered mental status and suspected seizures or to provide prognostic information. While the abbreviated duration of rEEGs inevitably reduces their sensitivity compared to cEEGs, their broad accessibility makes them an attractive modality for applying seizure risk scores such as the 2HELPS2B. Importantly, although the 2HELPS2B score has been repeatedly validated in cEEG cohorts, no prior study has systematically assessed its applicability to short-term rEEGs in critically ill patients. Our earlier findings demonstrated that rEEGs can predict short-term outcomes and identify seizure risk in patients in medical ICUs (MICUs) [13].

Building on these findings, previous studies consistently demonstrate the clinical utility of the 2HELPS2B score in predicting seizures among critically ill patients undergoing cEEG monitoring, including those with acute brain injury or other neurological emergencies [8,9,10,11]. These investigations confirmed that higher scores are strongly associated with increased seizure risk and have validated the score in diverse ICU and emergency settings. However, most of these studies focused exclusively on continuous EEGs, which are resource-intensive and not available in all hospitals. To date, no study has systematically applied the 2HELPS2B score to rEEGs in the MICU setting. Given that rEEGs are more widely accessible and often used to evaluate altered mental status or suspected seizures, this gap limits the translational impact of the 2HELPS2B score on broader clinical practice.

In this study, we evaluated the role of rEEGs in critical care by applying the 2HELPS2B score to interpret rEEG findings and assess its ability to predict epileptic seizures. Additionally, we examined whether rEEGs influence the prescription of antiseizure medications (ASMs) by MICU attending physicians and the association between ASM prescriptions and subsequent seizures in patients in the MICU. In this study, we attempt to (1) extend the clinical utility of the 2HELPS2B score to rEEGs in a relatively large MICU cohort; (2) identify an optimal cutoff score for predicting seizures and highlights its value for early risk stratification in critically ill patients; and (3) provide novel insights into the relationship between rEEG findings, seizure incidence, and ASM prescription patterns.

## 2. Materials and Methods

### 2.1. Study Design and Framework

This study followed a retrospective cohort design. The overall workflow consisted of the following steps: (1) identification of patients admitted to the MICU who underwent rEEG between October 2018 and March 2023, excluding those with epilepsy-related diagnoses or who were initially admitted to the neurological ICU; (2) collection of demographic and clinical data, including seizure occurrence and ASM prescriptions; (3) acquisition and interpretation of rEEGs in accordance with the ACNS terminology, with classification into six predefined EEG categories; (4) assignment of 2HELPS2B scores based on seizure history and EEG features; (5) assessment of outcomes, including seizure occurrence within 24 h and during the MICU stay and changes in ASM treatment; and (6) statistical analyses, including Fisher’s exact test or chi-square test for categorical variables and ROC analysis with the Youden index to determine the optimal cutoff value of the 2HELPS2B score.

### 2.2. Study Population and Data Collection

**Figure 1:**
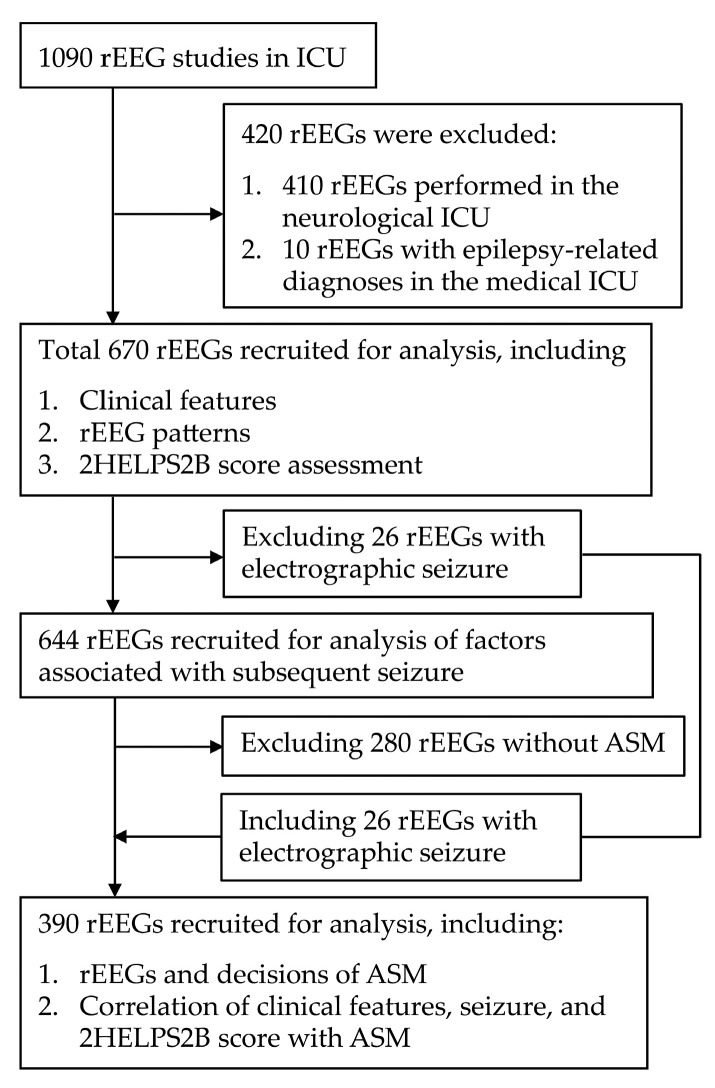
Flowchart of patient enrollment. rEEG, routine electroencephalography; ICU, intensive care unit; ASM, antiseizure medication.

This retrospective study analyzed patients who were admitted to the MICU of the index hospital and underwent rEEG between October 2018 and March 2023. After excluding 410 patients initially admitted to the neurological ICU and 10 patients with epilepsy-related diagnoses—such as convulsive status epilepticus or seizure clusters admitted to MICU—a total of 670 rEEG reports were recruited for analysis (Figure 1). The data collected included demographic information (age and sex), clinical indications for the rEEG, the interval between MICU admission and rEEG initiation, and seizure occurrence prior to or within 24 h after the rEEG, as well as during the overall MICU stay. Ethnicity was not systematically recorded in our hospital database; however, the patient population of our MICU predominantly consists of Han Chinese individuals, consistent with the regional demographic distribution in northern Taiwan.

The initiation of ASMs following the rEEG was also documented. All rEEGs were performed at the patient’s bedside in the MICU. The procedures were coordinated by MICU attending physicians with or without prior input from neurology consultants. The primary reasons for ordering rEEGs included evaluating unexplained alterations in consciousness, detecting possible seizure activity, and evaluating neurological prognosis, particularly following cardiac arrest and the return of spontaneous circulation.

### 2.3. Statement of Ethics

This study was conducted in accordance with the ethical principles of the Declaration of Helsinki. Ethical approval was obtained from the Institutional Review Board of Taipei Tzu Chi Hospital, New Taipei City, Taiwan (approval number: 13-IRB034). Owing to the retrospective nature of this study, the requirement for written informed consent was waived by the IRB in compliance with applicable national regulations. All data were collected from clinical records without influencing ongoing patient care. To ensure confidentiality and protect patient rights, only anonymized data were used in the analysis and reported in the manuscript, and no identifiable information was disclosed without explicit patient consent.

### 2.4. Indications for rEEGs

The indications for rEEGs were categorized into four groups: (1) seizure evaluation, (2) evaluation of unexplained altered consciousness, (3) follow-up evaluation of previously abnormal rEEG findings such as epileptiform discharges or abnormal background activities, and (4) prognostic evaluation. These indications were obtained from medical records prepared by the attending physician at the time of the rEEG request. The timing of the rEEGs was not predetermined by a fixed protocol but depended on the clinical judgment of the attending physicians.

### 2.5. Interpretation of EEG Patterns

All rEEGs in the MICU were recorded digitally by experienced technologists using a 21-electrode scalp configuration based on the International 10–20 system, in accordance with ACNS guidelines. A Nicolet vEEG system (VIASYS Healthcare, Middleton, WI, USA) was used, with a sampling rate of 250 Hz. The minimum recording duration for each rEEG was 15 min, and in most cases, recordings lasted 20–30 min. Recordings were primarily reviewed in the standard longitudinal bipolar montage, although alternative montages were applied when clinically indicated. A randomly selected general neurologist initially reported each rEEG shortly after the examination, and an experienced neurologist specializing in epilepsy subsequently reviewed each report retrospectively, using the latest edition of the standardized critical care EEG terminology proposed by the ACNS [6].

The rEEG findings were systematically categorized into six distinct groups: (1) nonspecific slow waves (NSWs), (2) suppression or burst suppression (SBS), (3) sporadic epileptiform discharges (SEDs), (4) rhythmic and periodic patterns (RPPs), (5) brief potentially ictal rhythmic discharges (BIRDs), and (6) electrographic seizures (ESz).

#### 2.5.1. NSWs

NSWs indicated the absence of epileptic discharges meeting ACNS criteria throughout the entire EEG recording, with only slow waves observed. Furthermore, suppression (defined as amplitude below 10 μV) or attenuation (amplitude above 10 μV but less than 50% of the background level) was only considered acceptable if present for under half of the total EEG recording time.

#### 2.5.2. SBS

SBS and burst-attenuation patterns were classified under a single category. Suppression or attenuation needed to be present for no less than half of the total EEG recording duration. Additionally, each burst had to persist for over 0.5 s and contain more than three distinct phases.

#### 2.5.3. SEDs

Spikes, polyspikes, or sharp waves that met the ACNS definition of SEDs yet did not align with the definitions of RPPs, ESz, or electroclinical seizures were classified as sporadic discharges, irrespective of the surrounding EEG context.

#### 2.5.4. RPPs

As defined by the ACNS guidelines, periodic discharges, rhythmic delta activity, and spike-and-wave or sharp-and-wave forms were collectively considered RPPs. Classification by topography included generalized, lateralized, bilateral independent, unilateral independent, and multifocal variants, depending on the regions in which the discharges were observed [6]. Based on the established classifications, the analysis encompassed patterns including lateralized periodic discharges (LPDs), bilateral independent periodic discharges (BIPDs), generalized periodic discharges (GPDs), lateralized rhythmic delta activity (LRDA), and generalized rhythmic delta activity (GRDA). The frequencies of RPPs were measured in 0.5 Hz increments.

#### 2.5.5. BIRDs

**Figure 2:**
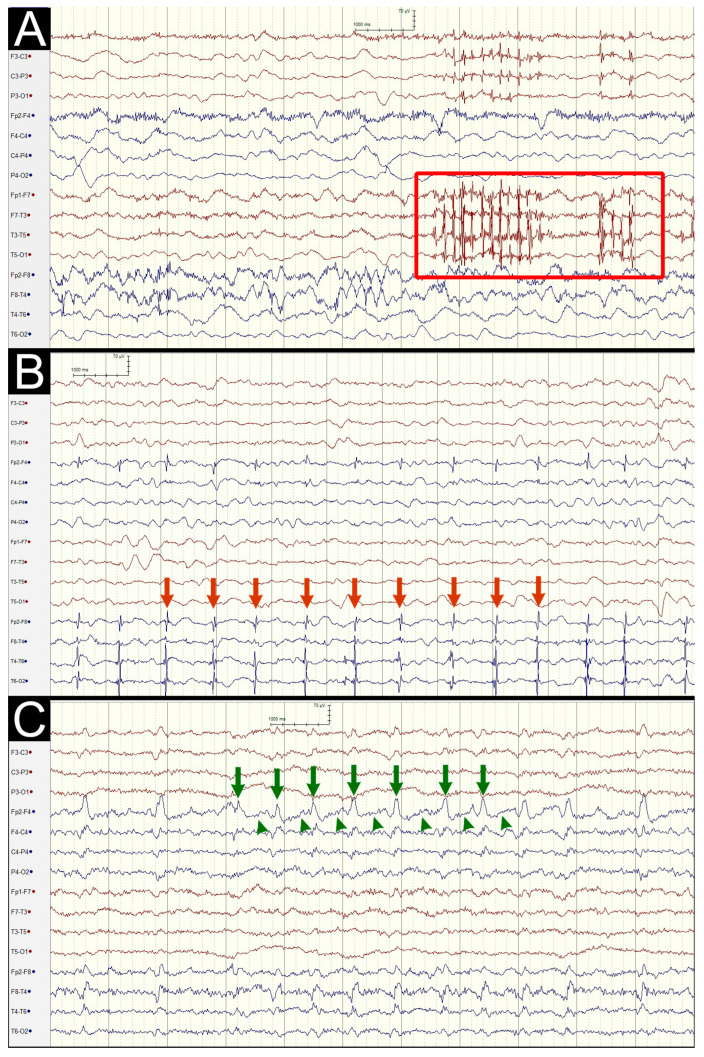
Three electroencephalography patterns: (**A**) 5 Hz spikes occurring in the left hemisphere with durations of 1.5 s and 0.5 s, a finding consistent with the features of brief potentially ictal rhythmic discharges (red box); (**B**) 1 Hz spikes (red arrows) occurring at regular intervals in the right hemisphere, a finding consistent with lateralized periodic discharges without plus features; and (**C**) 1.0–1.5 Hz sharp waves (green arrows) with small bursts of rapid activity at intervals (green arrowheads) occurred in the right hemisphere, which indicate lateralized periodic discharges with plus features.

BIRDs are characterized by focal or generalized rhythmic activity that exceeds 4 Hz and is composed of at least six waves occurring at consistent intervals, with durations ranging from 0.5 s to less than 10 s. These patterns differ from established normal variants or benign rhythms and are not considered components of burst-suppression or burst-attenuation patterns (Figure 2A).

#### 2.5.6. ESz

ESzs were diagnosed when epileptiform discharges averaged above 2.5 Hz for a duration of at least 10 s or when any pattern demonstrated clear evolution and persisted for a minimum of 10 s. Continuous ESzs lasting for 10 min or longer, or those that occupied 20% or more of any 60 min EEG recording, were categorized as electrographic status epilepticus and included as ESzs in our analysis [6].

### 2.6. HELPS2B Score

The 2HELPS2B score was developed by Struck et al. to stratify the risk of ESzs in hospitalized patients undergoing cEEG monitoring. This validated tool integrates five EEG features and one clinical variable (seizure history), yielding a total score of 0 to 7. According to the scoring rules, 2 points were assigned when BIRDs were present, while 1 point was given for LPDs, LRDA, or BIPDs, and an additional point was counted for SEDs. One point was also assigned when RPPs occurred at a frequency greater than 2 Hz or when “plus” features, defined as superimposed rhythmic, sharp, or fast activity on LPDs, LRDA, or BIPDs, were observed. A history of seizures was further scored with 1 point (Figure 2B,C).

The 2HELPS2B score is calculated within the first hour following the initiation of a cEEG (screening EEG) and stratifies patients into three categories, with a score of 0 corresponding to low risk (seizure probability < 5%), a score of 1 indicating medium risk (approximately 12%), and a score of 2 or higher designating high risk (probability > 25%). Based on these categories, the recommended minimum duration of cEEG monitoring is 1 h for low-risk patients, 12 h for medium-risk patients, and 24 h for those at high risk [8,9].

### 2.7. ASMs

We compared changes in ASM treatment before and at 1 day after the release of the rEEG report. ASM treatment decisions were classified into six categories: (1) “No change” indicated no modification in ASM type or dose, (2) “Reduction” indicated a reduced dose of the prescribed ASM, (3) “Incremented” indicated an increased dose of the prescribed ASM, (4) “Add-on” indicated the addition of a new ASM to the patient’s regimen, (5) “Switched” indicated the substitution of the original ASM with another, and (6) “New ASM” indicated the initiation of ASM treatment in patients who had not received ASMs before their rEEG.

### 2.8. Statistical Analysis

Given the skewed distribution of continuous variables, results are reported as medians with interquartile ranges (25th–75th percentiles). Categorical variables are expressed as counts and percentages (*n*, %). Group comparisons were primarily analyzed using Fisher’s exact test for 2 × 2 contingency tables, with the chi-square test employed for larger tables. Statistical significance was defined as a two-tailed *p* value < 0.05, and exact *p* values are reported where possible.

For categorical comparisons, odds ratios (ORs) with 95% confidence intervals (CIs) and *p* values were calculated using OpenEpi (www.openepi.com, version 3.01, accessed on 27 August 2025) based on 2 × 2 contingency tables. Continuous variables were compared using the Mann–Whitney U test, for which ORs are not applicable.

To evaluate the predictive performance of the 2HELPS2B score, receiver operating characteristic (ROC) curve analyses were conducted. The outcomes of interest were convulsive seizures occurring within 24 h of rEEG initiation and seizures before MICU discharge. ROC curves were generated using MedCalc (version 18.11.3; MedCalc Software, Mariakerke, Belgium). The area under the ROC curve (AUC) was reported, with 95% CIs calculated using DeLong’s method. The optimal cutoff values were determined by maximizing the Youden index. Confidence intervals for sensitivity, specificity, and the Youden index were estimated using bias-corrected and accelerated bootstrap resampling with 1000 iterations.

All statistical analyses were performed using SPSS (version 24; IBM, Armonk, NY, USA), MedCalc, and OpenEpi and reported in accordance with the SAMPL guidelines.

To visualize the distribution of univariate continuous variables (e.g., age and ICU-to-rEEG interval), we generated raincloud-style plots incorporating jittered individual data points, violin density plots, and boxplots representing medians, interquartile ranges (IQRs), and approximate 95% confidence intervals. These plots were created using Python (version 3.11; Python Software Foundation) with the seaborn (version 0.12) and matplotlib (version 3.7) libraries.

## 3. Results

During the 5-year study period, 560 patients underwent a total of 670 rEEGs. Among the 670 rEEGs, 323 (48%) were performed in male patients. Notably, 91 patients (16.0%) underwent multiple rEEGs, among whom 61 underwent two, 19 underwent three, 1 underwent four, and 2 underwent five. Among the 670 rEEGs, 337 (52.3%) were associated with clinical seizures, defined as seizures occurring during hospitalization and before MICU discharge.

**Figure 3:**
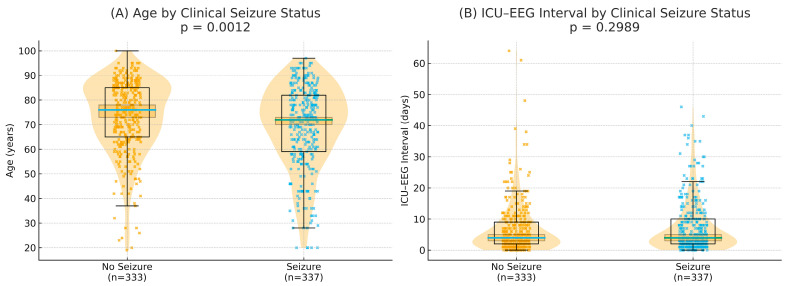
Raincloud plots of continuous variables by clinical seizure status. Each panel shows individual data points (jittered), the distribution (violin), the median (thick line), the interquartile range (box), and the 95% confidence interval of the median (shaded band). (**A**) Age: patients with seizures had a median age of 72 years (IQR 59–82, 95% CI 70–73) compared with 76 years (IQR 65–85, 95% CI 73–78) in those without seizures (*p* = 0.0012). (**B**) ICU–EEG interval: the median interval between ICU admission and rEEG was 4 days (IQR 2–10, 95% CI 3–5) in the seizure group versus 4 days (IQR 2–9, 95% CI 3–5) in the no-seizure group (*p* = 0.2989). EEG, electroencephalograph; ICU, intensive care unit.

Table 1 presents the clinical characteristics of the 670 rEEG reports. Seizure evaluation and unexplained altered consciousness were the two most common indications for requesting an rEEG and accounted for more than three-quarters of all rEEGs. The median interval from MICU admission to receiving an rEEG was 4 days (IQR 2–9). The distributions of age and ICU-to-rEEG intervals, stratified by clinical seizure status, are further illustrated in raincloud plots (Figure 3), which depict individual data points together with group medians, interquartile ranges, and 95% confidence intervals. Clinical seizures occurred in 213/259 patients (82.2%) receiving an rEEG for seizure evaluation and in 28/251 (11.2%) of the patients receiving an rEEG for altered consciousness. A total of 295/670 (44.0%) rEEGs were conducted in patients with a history of seizures. All clinical seizures coincided with a history of seizure (295/295, 100%). ASMs were prescribed to 333/670 patients (49.7%) before their rEEG and in 350/670 (52.2%) after. Among the patients with a history of seizures, 262/295 (88.8%) received ASM treatment before their rEEG. Among the patients to whom ASM treatment was administered before the rEEG, 262/333 (78.7%) had a history of seizure. The number of patients receiving ASM treatment increased from 333 before the rEEG to 350 after the rEEG, with a greater increase observed in the group that experienced clinical seizures.

Of the 670 rEEGs, 68 (10.1%) were used for prognostic assessment, particularly to evaluate neurological outcome in patients after cardiac arrest. These rEEGs were intended to support the prediction of long-term recovery potential, even if they were not directly related to seizure detection. As prognostic evaluation was not directly related to seizure detection, it was not included in the subsequent analysis.

Table 2 presents the distributions of rEEG patterns and 2HELPS2B scores. More than half of the rEEGs exhibited an NSW pattern. BIRDs and ESz were detected in only 0.4% (3/670) and 3.9% (26/670) of the rEEG reports, respectively. However, clinical seizures occurred in all rEEGs exhibiting BIRDs (3/3, 100%) and ESz (26/26, 100%), followed by RPPs (77.6%), SEDs (69.7%), SBSs (45.8%), and NSWs (35.7.0%). Among the patients whose rEEGs exhibited RPPs, clinical seizures were most frequent in those whose rEEGs exhibited GPD (17/19, 89.5%) and LPD (58/71, 81.7%) patterns. The incidence of clinical seizures across RPP frequencies ranging from 0.5 to 2.0 Hz varied between 70.3% and 80.9%. Regarding the individual 2HELPS2B score components, seizure history was most common (295/670, 44.0%), followed by the presence of LPDs, BIRDs, or LRDAs (80/670, 11.9%) and SEDs (76/670, 11.3%). The median 2HELPS2B score was significantly higher for patients who experienced clinical seizures (1, IQR 1–1) than for those who did not (0, IQR 0–0; *p* < 0.0001, Mann–Whitney U test). A positive 2HELPS2B score (≥1) was assigned for 347/670 (51.8%) rEEGs, and positive scores occurred significantly more often for the rEEGs of patients with clinical seizures (303/337, 89.9%) than for those without clinical seizures (44/333, 13.2%; *p* < 0.0001).

To evaluate the ability of rEEGs to predict clinical seizures, we compared clinical characteristics and EEG patterns between the patients who experienced and did not experience seizures both within 24 h of the initial rEEG and before MICU discharge (Table 3). Among the 26 rEEG reports exhibiting ESz patterns, 5 (1.9%) occurred in the 251 patients being evaluated for unexplained altered consciousness. Because the ESz pattern confirmed the occurrence of a clinical seizure, these 26 rEEG reports were excluded from further analysis. Hence, the 644 rEEG reports of the patients who experienced 311 clinical seizures are included in the comparisons presented in Table 3. Seizures occurred in 86/311 (27.7%) patients within 24 h of the initial rEEG and in 119/311 (38.3%) patients before MICU discharge. Approximately two-thirds of the patients for whom rEEGs were requested for seizure evaluation developed clinical seizures within 24 h (67.4%) of the rEEG or before MICU discharge (64.7%). Seizures occurred after 11 rEEGs were requested for unexplained altered consciousness. Including the five previously excluded ESz rEEG reports, seizures occurred after 16/252 (6.3%) rEEGs requested for unexplained altered consciousness. Among these, the seizure incidence before MICU discharge (11/119, 9.2%) was twice that within 24 h (4/86, 4.7%) of the initial rEEG. This finding suggests that repeated rEEGs may assist in the early detection of delayed-onset seizures after an initial rEEG. Clinical seizures continued to occur after rEEGs in 57/97 (58.8%) patients who had seizures before undergoing an rEEG. Among those who experienced seizures after the rEEG, 95/119 (79.8%) had received ASM treatment before the rEEG.

Our analysis revealed significant associations between the individual components and total scores of the 2HELPS2B scale and seizure occurrence after an rEEG (Table 3). All 86 rEEG reports associated with seizures within 24 h were assigned 2HELPS2B scores of ≥1. The seizure rate increased from 12.0% (26/218) for a score of 1 to 100% (5/5) for scores ≥4.

**Figure 4:**
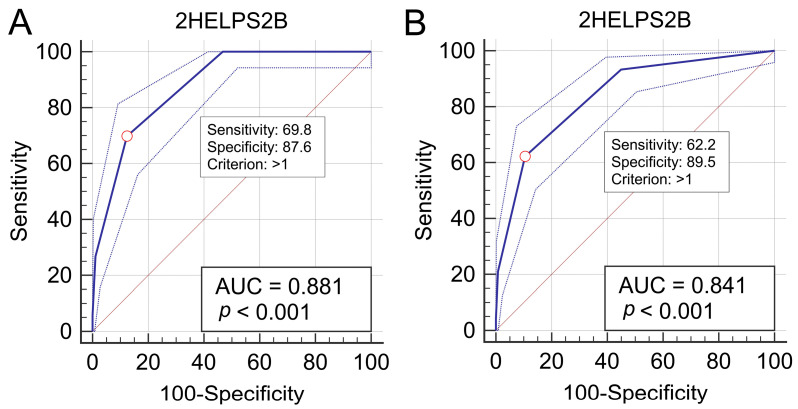
Receiver operating characteristic (ROC) curves for 2HELPS2B score. (**A**) Prediction of seizures within 24 h after rEEG initiation. (**B**) Prediction of seizures before MICU discharge. Shaded areas indicate 95% confidence bands estimated using bootstrap resampling (1000 iterations).

ROC curve analysis identified a 2HELPS2B score > 1 as the optimal cutoff for predicting seizures within 24 h of an initial rEEG, yielding an AUC of 0.881 (95% CI: 0.853–0.905; *p* < 0.001), a sensitivity of 69.8% (95% CI: 58.9–79.2), a specificity of 87.6% (95% CI: 84.6–90.3), and a Youden index of 0.574 (95% CI: 0.513–0.672) (Figure 4A). For predicting seizures before MICU discharge, the same cutoff provided an AUC of 0.841 (95% CI: 0.811–0.869; *p* < 0.001), a sensitivity of 62.2% (95% CI: 52.8–70.9), a specificity of 89.5% (95% CI: 86.6–92.0), and a Youden index of 0.517 (95% CI: 0.442–0.590) (Figure 4B). For data for which the number of intervals for each continuous variable is 1, “>1” is equivalent to “≥2”. For better clinical application, we selected 2HELPS2B ≥ 2 as the cutoff value.

The association between rEEG patterns and ASM treatment adjustments was analyzed among 390 rEEG reports (including the 26 with ESz patterns) performed during MICU hospitalization (Table 4). The patients whose ASMs were unchanged or reduced accounted for 233/390 (59.7%) of all ASM treatment decisions; their rEEGs most frequently exhibited NSW patterns. Incremented and add-on ASMs accounted for 95/390 (24.4%) treatments and were prescribed most frequently to patients whose rEEGs exhibited RPPs. New ASM initiation accounted for 56/390 (14.4%) treatments and was most frequently associated with SED and RPPs. ASMs were switched after 6/390 (1.5%) rEEGs. The overall correlation between rEEG patterns and ASM treatment adjustments was statistically significant (*p* < 0.0001, Fisher’s exact test). These data indicate that specific rEEG patterns, particularly RPPs, ESz, and BIRDs, were closely associated with subsequent aggressive therapeutic adjustments, although the interpretation of BIRDs is limited by the very small sample size.

We further examined the associations of clinical features, seizures, and the 2HELPS2B score with physicians’ ASM management decisions across 390 rEEG reports (Table 5). Clinical seizures occurred in 132/390 (33.8%) cases after rEEGs were obtained, with 103/132 (78.0%) occurring within 24 h. ASM adjustments were evenly distributed among the rEEGs requested for seizure evaluation, although the frequency of the switched and new ASM categories was lower than that of the other ASM categories. New ASMs were initiated after 56/390 (14.4%) rEEGs, predominantly in cases involving unexplained altered consciousness. Clinical seizures occurred more frequently in the add-on group, both within 24 h (48/60, 80.0%) of the rEEG and before MICU discharge (53/60, 88.3%). More aggressive ASM adjustments were observed in cases with 2HELPS2B scores ≥ 2; specifically, incremented ASMs were observed in 57% of cases, add-on ASMs in 67%, and switched ASMs in 67%. These data suggest that add-on therapy carries a higher risk of subsequent seizures compared with other escalated therapies.

## 4. Discussion

This study observed that seizure history and ASM use before undergoing an rEEG were associated with a higher incidence of seizures. Most patients who received ASMs before undergoing an rEEG had a history of seizures, and most with a history of seizures had previously received ASMs. Risk of seizure recurrence remains high in patients with a history of seizures who exhibit poor ASM compliance. However, recurrent seizures may also occur in patients receiving regular ASMs during severe illness. Accurate recognition of clinical seizures and timely ASM treatment are critical in managing severe illness, particularly in cases involving NCSs. Studies have reported that NCSs occur in 15% to 20% of patients in the ICU [2,4]. Struck et al. also reported that a history of seizures was associated with a higher incidence of NCSs or NCSE evident on cEEGs [7]. In this study, ESzs occurred in only 4% (26/670) of the rEEG reports. These 26 patients were excluded from the analysis of seizure prediction as the presence of an ESz pattern confirmed seizure occurrence, rendering further prediction unnecessary. Given that the 2HELPS2B score is specifically designed to predict seizures, it was not applicable for these patients. Following the detection of ESzs, all these patients received intravenous ASMs and were transferred to the neurological ICU for further care. As a result, their long-term prognosis was not tracked in this study. Among the remaining 644 reports, seizures occurred in 13% of the studied patients within 24 h of the rEEG and in 18% prior to MICU discharge. Most seizures were convulsive, likely because of the lower sensitivity of rEEGs in detecting epileptiform discharges compared with that of cEEGs [14]. NCSs may remain undetected if physicians fail to recognize subtle rhythmic facial or ocular twitches and no epileptiform discharges are evident on rEEGs. However, one study indicated that compared with rEEGs, cEEGs provide minimal additional information [14].

EEG features, specifically, lateralization, plus features, and higher RPP frequency, were associated with an increased incidence of seizures after an rEEG, and LPD was the predominant form of lateralized RPP. Studies have established a strong correlation between LPD and epilepsy. For example, Schraeder et al. reported seizures in 20 of 24 patients with LPD [15], and Baykan et al. reported that 37 of 45 patients with LPD experienced seizures during hospitalization, including 26 who experienced seizures for the first time [16]. Nevertheless, few studies have examined plus features in isolation. Fatima et al. reported a greater probability of seizures in patients with LPD and plus features than in those with LPD without plus features [17].

In this study, all three rEEGs exhibiting BIRD patterns were followed by clinical seizures within 24 h. BIRDs represent a distinct category of electroencephalographic abnormality that bridges interictal and ictal patterns. Although brief (<10 s), BIRDs are strongly associated with increased seizure risk across multiple patient groups, such as neonates, critically ill adults, and individuals with epilepsy who are not critically ill. One study has also suggested that the location of BIRDs may more accurately predict seizure origin than the location of other interictal epileptiform discharges [18].

Although it was originally developed to predict seizures from cEEG reports, as indicated in this study, the 2HELPS2B score can also be used to predict seizures on the basis of rEEG evaluations. Specifically, no seizures occurred within 24 h in patients with a score of 0. However, as the score increased from 1 to ≥4, the incidence of seizures increased from 12% to 100%. Higher scores consistently corresponded with increased seizure occurrence. A 2HELPS2B score ≥ 2 was the optimal cutoff for predicting seizures after rEEGs, with an area under the ROC curve ranging from 0.836 to 0.877. These findings indicate that the 2HELPS2B score reliably predicts convulsive seizures within 24 h after examination and throughout the MICU stay when applied to interpret the rEEGs of patients in the MICU.

Although our analysis identified 2HELPS2B > 1 as the optimal cutoff based on the Youden index, the score may also be interpreted in a dual clinical framework. A threshold of ≥1 may serve as a screening score, helping to identify patients at elevated risk who warrant closer monitoring or cEEG evaluation, particularly when preventive interventions carry minimal or no side effects. Conversely, a threshold of ≥2 with higher specificity may serve as a validation score, guiding preventive antiseizure treatments when the balance of potential benefits and adverse effects needs to be carefully considered. While this dual usage was not the primary focus of our study, our findings provide preliminary support for such an approach and suggest that further research is warranted.

To further contextualize these findings, we compared the 2HELPS2B score with other commonly used methods for seizure risk prediction in the ICU. Table 6 summarizes the advantages and disadvantages of different methods in terms of data sources, clinical applicability, predictive performance, and limitations. The rEEG-based 2HELPS2B score can be a more convenient method compared with cEEGs and can more reliably predict seizures compared to clinical features alone or EEG markers.

Among the patients who received ASM treatment, approximately 60% experienced no change or a reduction in dose after their rEEG. By contrast, approximately 40% received more aggressive seizure management through increased dosage, medication substitution, the addition of a new ASM, or the initiation of ASM therapy. Aggressive ASM adjustments were administered in 79% of cases with RPPs, 67% of cases with BIRDs, and 81% of cases with ESzs. These EEG patterns typically indicate inadequate seizure control and increased seizure risk. However, even with aggressive ASM intervention, seizure incidence within 24 h after an rEEG remained high in the patients whose rEEGs exhibited these patterns. This persistence may result from delayed ASM adjustments, delayed interpretation of rEEG findings, inadequate detection of abnormal EEG activity by neurologists, or delayed responses by ICU physicians to rEEG reports.

Seizure management in critically ill patients remains complex and involves ASM pharmacokinetics, comorbidities, drug interactions, and physiological factors such as renal and hepatic function. This complexity is compounded by a lack of uniform treatment guidelines for seizures. Recommendations must consider the comprehensive clinical context to formulate individualized treatment plans [19,20]. Notably, timely diagnosis and appropriate intervention are critical [21]. In the present study, unchanged or reduced ASM treatment typically followed clinical improvement or improved rEEG findings. However, ASM dose reductions also occurred in some patients with a deteriorating medical condition.

The influence of aggressive ASM adjustments on seizure control after rEEGs varied. Dose increments typically addressed inadequate serum drug levels or subtherapeutic dosing, and add-on decisions were made when the original ASM failed to control seizures at therapeutic doses. Decisions to switch medications were made under similar circumstances, often compounded by adverse effects or drug interactions associated with the original ASM. Seizure type differences also contributed to switching decisions. For patients initially considered seizure-free, initiating a new ASM was the most critical intervention. New ASM initiation occurred most frequently in cases involving unexplained altered consciousness (Table 5), indicating the importance of rEEGs in diagnosing patients with this condition. Additionally, our results suggest that repeated rEEG examinations are required to evaluate treatment efficacy and guide ASM adjustments in patients unresponsive to initial therapy.

Brodie et al. reported that 47% of previously drug-naïve patients responded to their first ASM, 13% achieved seizure freedom with a second ASM, and 1% became seizure-free after a third monotherapy [22]. These findings suggest that patients without prior ASM exposure have the highest probability of achieving seizure control with their initial treatment. However, seizure management in critically ill patients is complex, particularly in those with a history of seizures. Aggressive ASM treatment was required in 39% of cases in this study, with 16% requiring more intensive intervention through add-on or modified ASM strategies.

Monotherapy has traditionally been the preferred approach to managing epilepsy because of its low side effects, absence of drug interactions, superior adherence, and lower cost [23,24]. However, this traditional perspective has evolved, and combination therapy is no longer regarded as inferior because newer ASMs offer enhanced tolerability and reduced drug interactions [25,26,27]. Although some studies have reported increased tolerability after switching therapies, these advantages do not consistently translate to enhanced efficacy [28,29]. In this study, the add-on treatment group experienced a significantly higher incidence of seizures than the incrementing and switching groups did. This result contrasts with those of studies that indicated the noninferiority of add-on therapy and underscores a need to reevaluate treatment strategies in certain patient populations. Many patients also received subtherapeutic ASM doses prescribed by ICU physicians. Most decisions to add or switch ASMs were made following neurology consultations. In several cases in which the ICU physicians independently prescribed add-on ASMs, the doses remained below the recommended levels.

This study has several limitations. First, the rEEG patterns were interpreted retrospectively according to the latest ACNS guidelines. We did not assess how these retrospective interpretations compared with the original reports that informed clinical decisions, which may partly explain why some patients exhibiting RPP or NCS patterns on their rEEGs did not receive ASMs after the initial rEEG. Second, ASM treatment and dosing decisions made by ICU physicians before neurology consultations were not accessible, and the reasons for delays in adjusting ASM treatment after the rEEGs remain unknown. Therefore, the association between seizure occurrence within 24 h of the initial rEEG and subsequent ASM adjustment may be indirect. Third, because cEEGs were unavailable at the study hospital, comparisons between seizure detection rates for rEEGs and cEEGs were impossible. Additionally, many hospitals do not provide cEEGs in the MICU. Therefore, the clinical benefits of rEEGs demonstrated in this study have crucial implications.

Furthermore, some EEG patterns were detected only rarely in our cohort. For example, BIRDs were observed in only three rEEG reports, which limits the ability to draw definitive conclusions about their predictive value. This low frequency most likely reflects the characteristics of our MICU population rather than a methodological issue, as all rEEGs were interpreted according to standardized ACNS terminology by experienced neurologists. Nevertheless, the small number of BIRD cases restricts statistical power, and validation in larger multicenter cohorts will be required to further clarify their role in routine EEGs.

This study was conducted in a single medical center, and all patients were adults admitted to a medical ICU, which may limit the extrapolation of our findings to pediatric populations or to patients treated in specialized neurological ICUs. The representativity and generalizability of our results warrant further studies among different hospitals. Nevertheless, the patient distribution in our MICU is comparable to that typically observed in other adult medical ICUs, suggesting that our results may reasonably reflect broader critically ill populations. Further large multicenter studies are necessary to validate these results. Finally, future prospective studies can provide more convincing data to evaluate whether incorporating the 2HELPS2B score into rEEG interpretation helps to guide ASM management strategies and improve long-term patient outcomes. Direct comparisons of rEEG- and cEEG-based seizure prediction would further clarify the relative utility of these approaches in different clinical settings.

## 5. Conclusions

The results of rEEGs provide a critical reference for seizure management. The 2HELPS2B score effectively predicts seizure occurrence from rEEG evaluations, with a score of ≥2 being the optimal cutoff for guiding ASMs treatment. Repeated rEEGs are recommended for patients with an initial rEEG demonstrating ESz or RPPs to enhance seizure monitoring. Timely ASM adjustment on the basis of rEEG findings is essential to effective seizure control. Integrating rEEG-based 2HELPS2B scoring into routine MICU practice can improve risk stratification and guide individualized ASM strategies, even in settings where cEEGs are not readily available. Future multicenter and prospective studies are warranted to validate these results and to determine whether incorporating the 2HELPS2B score into rEEG interpretation can ultimately improve long-term patient outcomes.

## Figures and Tables

**Table 1:** Clinical features of patients in 670 rEEG reports.

Characteristic	Total (*n* = 670)	Clinical Seizures		OR (95% CI)	*p* Value
		Yes (*n* = 337)	No (*n* = 333)		
Reason for rEEG					<0.0001
Seizure evaluation	259 (38.7%)	213 (63.2%)	46 (13.8%)	10.72 (7.32–15.70)	
Unexplained altered consciousness	251 (37.5%)	28 (8.3%)	223 (67%)	0.04 (0.03–0.07)	
Previous abnormal rEEG	92 (13.7%)	63 (18.7%)	29 (8.7%)	2.41 (1.51–1.85)	
Prognosis evaluation	68 (10.1%)	33 (9.8%)	35 (10.5%)	0.92 (0.56–1.53)	
Prior seizure	295 (44%)	295 (87.5%)	0 (0%)	-	<0.0001
MICU to rEEG (days)	4 (2–9)	4 (2–10)	4 (2–9)	-	0.2989
ASMs before rEEG	333 (49.7%)	282 (83.7%)	51 (15.3%)	28.35 (18.72–42.94)	<0.0001
ASMs after rEEG	350 (52.2%)	296 (87.8%)	54 (16.2%)	37.30 (24.08–57.78)	<0.0001

Data are presented as medians (interquartile ranges) for continuous variables and n (%) for categorical variables. Continuous variables were compared using the Mann–Whitney U test. Categorical variables were compared using Fisher’s exact test or chi-square test, as appropriate, and odds ratios (ORs) with 95% confidence intervals (CIs) are reported alongside *p* values. ASMs, antiseizure medications; MICU, medical intensive care unit; rEEG, routine electroencephalograph.

**Table 2:** Distribution of rEEG patterns and 2HELPS2B scores of 670 rEEG reports.

Characteristics	Total (*n* = 670)	Clinical Seizure		OR (95% CI)	*p* Value
		Yes (*n* = 337)	No (*n* = 333)		
rEEG patterns					
NSW	375 (56%)	134 (39.8%)	241 (72.4%)	0.25 (0.18–0.35)	<0.0001
SBS	83 (12%)	38 (11%)	45 (14%)	0.81 (0.51–1.29)	0.4126
SED	76 (11.3%)	53 (15.7%)	23 (6.9%)	2.52 (1.50–4.21)	0.0004
RPPs	107 (16%)	83 (24.6%)	24 (7.2%)	4.21 (2.59–6.82)	<0.0001
RPPs					
LPD	*71 (10.6%)*	*58 (17.2%)*	*13 (3.9%)*	*5.11 (2.75–9.54)*	*<0.0001*
BIPD	*4 (0.6%)*	*1 (0.3%)*	*3 (0.9%)*	*0.33 (0.03–3.16)*	*0.3709*
LRDA	*5 (0.7%)*	*3 (0.9%)*	*2 (0.6%)*	*1.49 (0.15–8.95)*	*>0.9999*
GPD	*19 (2.8%)*	*17 (5.0%)*	*2 (0.6%)*	*8.79 (2.02–38.36)*	*0.0006*
GRDA	*8 (1.2%)*	*4 (1.2%)*	*4 (1.2%)*	*0.99 (0.25–3.98)*	*>0.9999*
RPP frequency (Hz)					
0.5	*27 (4%)*	*19 (5.6%)*	*8 (2.4%)*	*2.43 (1.05–5.62)*	*0.0477*
1	*43 (6.4%)*	*35 (10.4%)*	*8 (2.4%)*	*4.71 (2.15–10.31)*	*0.0001*
1.5	*21 (3.1%)*	*17 (5%)*	*4 (1.2%)*	*4.37 (1.46–13.13)*	*0.0064*
2.0	*16 (2.4%)*	*12 (3.6%)*	*4 (1.2%)*	*3.04 (0.97–9.51)*	*0.0734*
BIRDs	3 (0.4%)	3 (0.9%)	0 (0%)	-	0.2489
ESz	26 (3.9%)	26 (7.7%)	0 (0%)	-	<0.0001
2HELPS2B items					
Frequency > 2Hz (score = 1)	17 (2.5%)	13 (3.9%)	4 (1.2%)	3.30 (1.07–10.23)	0.0463
SED (score = 1)	76 (11.3%)	53 (15.7%)	23 (6.9%)	2.52 (1.50–4.21)	0.0004
LPD/BIPD/LRDA (score = 1)	80 (11.9%)	62 (18.4%)	18 (5.4%)	3.95 (2.28–6.83)	<0.0001
Plus features (score = 1)	37 (5.5%)	32 (9.5%)	5 (1.5%)	6.88 (2.65–17.89)	<0.0001
Prior seizure (score = 1)	295 (44%)	295 (87.5%)	0 (0%)	-	<0.0001
BIRDs (score = 2)	3 (0.4%)	3 (0.9%)	0 (0%)	-	0.2489
Median 2HELPS2B score	0 (0–1)	1 (1–1)	0 (0–0)	-	<0.0001
2HELPS2B score ≥1	347 (51.8%)	303 (90%)	44 (13.2%)	58.53 (84.64–92.11)	<0.0001

Data are presented as medians (IQRs) for continuous variables and *n* (%) for categorical variables. Continuous variables were compared using the Mann–Whitney U test. Categorical variables were compared using Fisher’s exact test or chi-square test, as appropriate, and odds ratios (ORs) with 95% confidence intervals (CIs) are reported alongside *p* values. BIPD, bilateral independent periodic discharge; BIRDs, brief potentially ictal rhythmic discharges; ESz, electrographic seizures; GPD, generalized periodic discharge; GRDA, generalized rhythmic delta activity; LPD, lateralized periodic discharge; LRDA, lateralized rhythmic delta activity; NSW, nonspecific slow wave; rEEG, routine electroencephalography; RPPs, rhythmic and periodic patterns; SBS, suppression or burst suppression; SED, sporadic epileptiform discharge.

**Table 3:** Comparison of factors associated with subsequent seizure after rEEG in 644 rEEG reports, excluding electrographic seizure.

Characteristics	Seizure Within 24 h				Seizure Before Leaving MICU			
	Y (*n* = 86)	N (*n* = 558)	OR (95%CI)	*p* Value	Y (*n* = 119)	N (*n* = 525)	OR (95% CI)	*p* Value
Reason for rEEG				<0.0001				<0.0001
Seizure evaluation (*n* = 247)	58 (67.4%)	189 (33.9%)	4.04 (2.49–6.56)		77 (64.7%)	170 (32.4%)	3.83 (2.52–5.81)	
Unexplained altered consciousness (*n* =246)	4 (4.7%)	242 (43.4%)	0.06 (0.02–0.18)		11 (9.2%)	235 (44.7%)	0.13 (0.07–0.24)	
Previous abnormal rEEG (*n* = 87)	16 (18.6%)	71 (12.7%)	1.57 (0.86–2.85)		21 (17.6%)	66 (12.6%)	0.60 (0.35–1.05)	
Prognostic evaluation (*n* = 64)	8 (9.3%)	56 (10%)	0.92 (0.42–2.00)		10 (8.4%)	54 (10.3%)	0.80 (0.39–1.62)	
Clinical seizures before rEEG (*n* = 173)	53 (61.6%)	120 (21.5%)	5.86 (3.63–9.47)	<0.0001	70 (58.8%)	103 (19.6)	5.85 (3.83–8.94)	<0.0001
ASM before rEEG (*n* = 317)	73 (84.9%)	244 (43.7)	7.23 (3.91–13.34)	<0.0001	95 (79.8%)	222 (42.3%) (42.3%)<0.000	5.40 (3.34–8.73)	<0.0001
2HELPS2B items								
Frequency > 2Hz (*n* = 16)	8 (9.3%)	8 (1.4%)	7.05 (2.57–19.33)	0.0004	9 (7.6%)	7 (1.3%)	6.06 (2.21–16.60)	0.0007
SED (n = 76)	19 (22.1%)	57 (10.2%)	2.49 (1.40–4.45)	0.0034	24 (20.2%)	52 (9.9%)	2.30 (1.35–3.91)	0.0041
LPD/BIPD/LRDA (*n* = 80)	36 (41.9%)	44 (7.9%)	8.41 (4.96–14.25)	<0.0001	45 (37.8%)	35 (6.7%)	8.51 (5.14–14.10)	<0.0001
Plus features (*n* = 37)	26 (30.2%)	11 (2.0%)	21.55 (10.14–45.78)	<0.0001	28 (23.5%)	9 (1.7%)	17.64 (8.06–38.61)	<0.0001
Prior seizure (*n* = 295)	79 (91.9%)	216 (38.7)	17.87 (8.10–39.43)	<0.0001	103 (86.6%)	192 (36.6%)	11.17 (6.41–19.46)	<0.0001
BIRDs	3 (3.5%)	0 (0%)	-	0.0023	3 (2.5%)	0 (0%)	-	0.0062
2HELPS2B score				<0.0001				0.0069
=0 (*n* = 297)	0 (0%)	297 (53.2%)	-		8 (6.7%)	289 (55%)	0.06 (0.03–0.12)	
=1 (*n* = 218)	26 (30.2%)	192 (34.4%)	0.83 (0.50–1.35)		37 (31.1%)	181 (34.5%)	0.86 (0.56–1.32)	
=2 (*n* = 100)	37 (43%)	63 (11.3%)	5.93 (3.60–9.79)		49 (41.2%)	51 (9.7%)	6.51 (4.09–10.36)	
=3 (*n* = 24)	18 (20.9%)	6 (1.1%)	24.35 (9.35–63.45)		20 (16.8%)	4 (0.8%)	26.31 (8.81–78.63)	
≥4 (*n* = 5)	5 (5.8%)	0 (0%)	-		5 (4.2%)	0 (0%)	-	

Data are expressed as *n* (%) or median (IQR) values. Categorical comparisons were analyzed using Fisher’s exact test or chi-square test as appropriate, and ORs with 95% CIs are reported in addition to *p* values. The Mann–Whitney U test was applied for comparisons of 2HELPS2B scores. ASM, antiseizure medicine; BIRDs, brief potentially ictal rhythmic discharges; BIPD, bilateral independent periodic discharge; LPD, lateralized periodic discharge; LRDA, lateralized rhythmic delta activity; NSW, nonspecific slow wave; rEEG, routine electroencephalography; SED, sporadic epileptiform discharge.

**Table 4:** Correlation between rEEG results and physician decisions regarding antiseizure medication treatment in 390 rEEG cases.

	No Change (*n* = 196)	Reduction (*n* = 37)	Incremented (*n* = 35)	Add-on (*n* = 60)	Switched (*n* = 6)	New ASM (*n* = 56)	*p*-Value
rEEG finding							<0.0001
NSW (*n* =158)	105 (53.6%)	30 (81.1%)	7 (20.0%)	5 (8.3%)	1 (16.7%)	10 (17.9%)	
SED (*n* = 63)	27 (13.8%)	4 (10.8%)	10 (28.6%)	6 (10.0%)	1 (16.7%)	15 (26.8%)	
RPPs (*n* = 95)	22 (11.2%)	1 (2.7%)	15 (42.9%)	35 (58.3%)	3 (50.0%)	19 (33.9%)	
SBS (*n* = 45)	34 (17.3%)	2 (5.4%)	1 (2.9%)	4 (6.7%)	1 (16.7%)	3 (5.4%)	
BIRDs (*n* = 3)	1 (0.5%)	0 (0%)	0 (0%)	2 (3.3%)	0 (0%)	0 (0%)	
ESz (*n* = 26)	7 (3.6%)	0 (0%)	2 (5.7%)	8 (13.3%)	0 (0%)	9 (16.1%)	

Data are expressed as *n* (%). Categorical variables were analyzed using chi-square test, and ORs with 95% CIs are reported alongside *p* values. ASM, antiseizure medication; BIRDs, brief potentially ictal rhythmic discharges; ESz, electrographic seizures; NSW, nonspecific slow wave; rEEG, routine electroencephalography; RPPs, rhythmic and periodic patterns; SED, sporadic epileptiform discharge; SBS, suppression or burst suppression.

**Table 5:** Correlations of clinical features, seizure occurrence, and 2HELPS2B scores with antiseizure medications adjusted after rEEGs in 390 rEEG reports.

Characteristics	No Change (*n* = 196)	Reduction (*n* = 37)	Incremented (*n* = 35)	Add-on (*n* = 60)	Switched (*n* = 6)	New ASM (*n* = 56)	*p*-Value
Reason for rEEG							<0.0001
Seizure evaluation	109 (56%)	22 (59%)	23 (66%)	40 (67%)	2 (33%)	19 (34%)	
Unexplained altered consciousness	22 (11%)	5 (14%)	2 (6%)	2 (3%)	0 (0%)	28 (50%)	
Previous abnormal rEEG	41 (21%)	9 (24%)	7 (20%)	9 (15%)	2 (33%)	4 (7%)	
Prognosis evaluation	24 (12%)	1 (3%)	3 (9%)	9 (15%)	2 (33%)	5 (9%)	
Seizure within 24 h (*n* = 103)	21 (11%)	2 (5%)	14 (40%)	48 (80%)	2 (33%)	16 (29%)	<0.0001
Seizure before leaving MICU (*n* = 132)	39 (20%)	2 (5%)	16 (46%)	53 (88%)	2 (33%)	20 (36%)	<0.0001
2HELPS2B score ≥ 2 (*n* = 126)	46 (23%)	4 (11%)	20 (57%)	40 (67%)	4 (67%)	12 (21%)	<0.0001

Data are expressed as *n* (%). Categorical variables were analyzed using chi-square test, and ORs with 95% CIs are reported alongside *p* values. ASM, antiseizure medication; rEEG, routine electroencephalography.

**Table 6:** Comparison of different methods for seizure risk prediction in critically ill patients.

Method	Data Source	Clinical Applicability	Predictive Performance	Limitations
2HELPS2B (rEEG, present study)	Routine EEG (≤1 h), seizure history	Widely applicable in MICU; resource-efficient	Score ≥ 2 predicts post-rEEG seizures	Validation limited to single center; small number of BIRD cases
2HELPS2B (cEEG) [9]	Continuous EEG (12–24 h+)	Strong predictive value; validated in ICUs and EDs	Higher scores associated with imminent seizures	Requires cEEG; limited availability; costly
Clinical features alone [1]	History, bedside examination	Universally available	Low sensitivity for nonconvulsive seizures	Cannot reliably stratify seizure risk without EEG
EEG markers (e.g., RPP, ESz) [4]	EEG interpretation alone	Useful when specific patterns are present	Certain patterns are strongly associated with seizures; lack of standardized scoring system; inter-rater variability	

cEEG, continuous electrography; ESz, electrographic seizures; rEEG, routine electroencephalography; RPPs, rhythmic and periodic patterns.

## Data Availability

The data presented in this study are available from the corresponding author on request.

## References

[1] Benbadis S.R., Beniczky S., Bertram E., MacIver S., Moshé S.L. (2020). The role of EEG in patients with suspected epilepsy. Epileptic Disord..

[2] Oddo M., Carrera E., Claassen J., Mayer S.A., Hirsch L.J. (2009). Continuous electroencephalography in the medical intensive care unit. Crit. Care Med..

[3] Scheuer M.L. (2002). Continuous EEG monitoring in the intensive care unit. Epilepsia.

[4] Claassen J., Mayer S.A., Kowalski R.G., Emerson R.G., Hirsch L.J. (2004). Detection of electrographic seizures with continuous EEG monitoring in critically ill patients. Neurology.

[5] Hill C.E., Blank L.J., Thibault D., Davis K.A., Dahodwala N., Litt B., Willis A.W. (2019). Continuous EEG is associated with favorable hospitalization outcomes for critically ill patients. Neurology.

[6] Hirsch L.J., Fong M.W.K., Leitinger M., LaRoche S.M., Beniczky S., Abend N.S., Lee J.W., Wusthoff C.J., Hahn C.D., Westover M.B. (2021). American Clinical Neurophysiology Society’s Standardized Critical Care EEG Terminology: 2021 Version. J. Clin. Neurophysiol..

[7] Struck A.F., Ustun B., Ruiz A.R., Lee J.W., LaRoche S.M., Hirsch L.J., Gilmore E.J., Vlachy J., Haider H.A., Rudin C. (2017). Association of an Electroencephalography-Based Risk Score With Seizure Probability in Hospitalized Patients. JAMA Neurol..

[8] Struck A.F., Rodriguez-Ruiz A.A., Osman G., Gilmore E.J., Haider H.A., Dhakar M.B., Schrettner M., Lee J.W., Gaspard N., Hirsch L.J. (2019). Comparison of machine learning models for seizure prediction in hospitalized patients. Ann. Clin. Transl. Neurol..

[9] Struck A.F., Tabaeizadeh M., Schmitt S.E., Ruiz A.R., Swisher C.B., Subramaniam T., Hernandez C., Kaleem S., Haider H.A., Cissé A.F. (2020). Assessment of the Validity of the 2HELPS2B Score for Inpatient Seizure Risk Prediction. JAMA Neurol..

[10] Moffet E.W., Subramaniam T., Hirsch L.J., Gilmore E.J., Lee J.W., Rodriguez-Ruiz A.A., Haider H.A., Dhakar M.B., Jadeja N., Osman G. (2020). Validation of the 2HELPS2B Seizure Risk Score in Acute Brain Injury Patients. Neurocrit. Care..

[11] Nonaka M., Neshige S., Ono N., Yamada H., Takebayashi Y., Ishibashi H., Aoki S., Yamazaki Y., Shishido T., Agari D. (2024). Clinical manifestations and outcomes associated with a high 2HELPS2B score in patients with acute impaired consciousness. J. Neurol. Sci..

[12] Waak M., Laing J., Nagarajan L., Lawn N., Harvey A.S. (2023). Continuous electroencephalography in the intensive care unit: A critical review and position statement from an Australian and New Zealand perspective. Crit. Care Resusc..

[13] Hsiao C.-L., Chen P.-Y., Chen I.-A., Lin S.-K. (2024). The Role of Routine Electroencephalography in the Diagnosis of Seizures in Medical Intensive Care Units. Diagnostics.

[14] Elmer J., Coppler P.J., Solanki P., Westover M.B., Struck A.F., Baldwin M.E., Kurz M.C., Callaway C.W. (2020). Sensitivity of Continuous Electroencephalography to Detect Ictal Activity After Cardiac Arrest. JAMA Netw. Open.

[15] Schraeder P.L., Singh N. (1980). Seizure disorders following periodic lateralized epileptiform discharges. Epilepsia.

[16] Baykan B., Kinay D., Gökyigit A., Gürses C. (2000). Periodic lateralized epileptiform discharges: Association with seizures. Seizure.

[17] Fatima S., Sun M., Gjini K., Struck A.F. (2022). Association Between Lateralized Periodic Discharge Amplitude and Seizure on Continuous EEG Monitoring in Patients With Structural Brain Abnormality in Critical Illness. Front. Neurol..

[18] Yoo J.Y. (2022). BIRDs (Brief Potentially Ictal Rhythmic Discharges) watching during EEG monitoring. Front. Neurol..

[19] Almohaish S., Cook A.M., Brophy G.M., Rhoney D.H. (2023). Personalized antiseizure medication therapy in critically ill adult patients. Pharmacotherapy.

[20] Farrokh S., Tahsili-Fahadan P., Ritzl E.K., Lewin J.J., Mirski M.A. (2018). Antiepileptic drugs in critically ill patients. Crit. Care.

[21] Allen B., Vespa P.M. (2019). Antiseizure medications in critical care: An update. Curr. Opin. Crit. Care.

[22] Brodie M.J., Kwan P. (2002). Staged approach to epilepsy management. Neurology.

[23] Kwan P., Brodie M.J. (2006). Combination therapy in epilepsy: When and what to use. Drugs.

[24] Guberman A. (1998). Monotherapy or polytherapy for epilepsy?. Can. J. Neurol. Sci..

[25] Stephen L.J., Brodie M.J. (2012). Antiepileptic drug monotherapy versus polytherapy: Pursuing seizure freedom and tolerability in adults. Curr. Opin. Neurol..

[26] Semah F., Thomas P., Coulbaut S., Derambure P. (2014). Early add-on treatment vs alternative monotherapy in patients with partial epilepsy. Epileptic Disord..

[27] Omar H.R., Mohamedy E.A.E.-G., El-Saeed M.A., Mohamed A.S. (2024). A Comparative Study between Mono Antiepileptic Therapy and Poly Antiepileptic Therapy regarding Quality of Life in Adolescents with Epilepsy. Benha Med. J..

[28] Wang X., He R., Zeng Q., Wang Y., Zhu P., Bao Y., Du Y., Shen J., Zheng R., Xu H. (2019). Substitution has better efficacy than add-on therapy for patients with focal epilepsy after their first antiepileptic drug treatments fail. Seizure.

[29] Canevini M.P., De Sarro G., Galimberti C.A., Gatti G., Licchetta L., Malerba A., Muscas G., La Neve A., Striano P., Perucca E. (2010). Relationship between adverse effects of antiepileptic drugs, number of coprescribed drugs, and drug load in a large cohort of consecutive patients with drug-refractory epilepsy. Epilepsia.

